# Differences in access to virtual and in-person primary care by race/ethnicity and community social vulnerability among adults diagnosed with COVID-19 in a large, multi-state health system

**DOI:** 10.1186/s12913-022-07858-x

**Published:** 2022-04-15

**Authors:** Diana J. Govier, Hannah Cohen-Cline, Katherine Marsi, Sarah E. Roth

**Affiliations:** Center for Outcomes Research and Education (CORE), Providence, 5211 NE Glisan Street, Portland, OR 97213 USA

**Keywords:** Telehealth, Primary care, Disparities, Race, Ethnicity, Social vulnerability, Outpatient care

## Abstract

**Background:**

Research exploring telehealth expansion during the COVID-19 pandemic has demonstrated that groups disproportionately impacted by COVID-19 also experience worse access to telehealth. However, this research has been cross-sectional or short in duration; geographically limited; has not accounted for pre-existing access disparities; and has not examined COVID-19 patients. We examined virtual primary care use by race/ethnicity and community social vulnerability among adults diagnosed with COVID-19 in a large, multi-state health system. We also assessed use of in-person primary care to understand whether disparities in virtual access may have been offset by improved in-person access.

**Methods:**

Using a cohort design, electronic health records, and Centers for Disease Control and Prevention Social Vulnerability Index, we compared changes in virtual and in-person primary care use by race/ethnicity and community social vulnerability in the year before and after COVID-19 diagnosis. Our study population included 11,326 adult patients diagnosed with COVID-19 between March and July 2020. We estimated logistic regression models to examine likelihood of primary care use. In all regression models we computed robust standard errors; in adjusted models we controlled for demographic and health characteristics of patients.

**Results:**

In a patient population of primarily Hispanic/Latino and non-Hispanic White individuals, and in which over half lived in socially vulnerable areas, likelihood of virtual primary care use increased from the year before to the year after COVID-19 diagnosis (3.6 to 10.3%); while in-person use remained stable (21.0 to 20.7%). In unadjusted and adjusted regression models, compared with White patients, Hispanic/Latino and other race/ethnicity patients were significantly less likely to use virtual care before and after COVID-19 diagnosis; Hispanic/Latino, Native Hawaiian/Pacific Islander, and other race/ethnicity patients, and patients living in socially vulnerable areas were also significantly less likely to use in-person care during these time periods.

**Conclusions:**

Newly expanded virtual primary care has not equitably benefited individuals from racialized groups diagnosed with COVID-19, and virtual access disparities have not been offset by improved in-person access. Health systems should employ evidence-based strategies to equitably provide care, including representative provider networks; targeted, empowering outreach; co-developed culturally and linguistically appropriate tools and technologies; and provision of enabling resources and services.

## Background

In the United States (U.S.) to date, there have been over 870,000 deaths related to coronavirus disease 2019 (COVID-19) and more than 73 million cases since January 2020 [1]. Unsurprisingly, given the well-documented connection between systemic inequities and structural racism, and the disparate distribution of health burden in the U.S. [2–9], COVID-19 has had a disproportionate impact on racialized groups and socially vulnerable communities. Greater rates of COVID-19 cases, hospitalizations, and deaths have been reported among Hispanic/Latino, American Indian/Alaska Native, Asian, Black/African American, and Native Hawaiian/Pacific Islander populations than among White, non-Hispanic populations. Socially vulnerable communities characterized by factors such as increased poverty and crowded housing have also experienced greater rates of COVID-19 infections and mortality [10–16].

Symptoms of COVID-19 are highly variable in both extent and type, and can affect multiple organ systems [17–19]. As many as one in three patients with COVID-19 report symptoms persisting beyond 4 weeks [17, 20], and following even mild cases some patients experience symptoms lasting months after COVID-19 onset, so called COVID-19 long-haulers [21]. Given this, appropriate post-COVID-19 infection care includes monitoring for persistent post-COVID-19 conditions and sequelae and treating those that arise [22]. Unfortunately, healthcare providers and researchers are becoming increasingly concerned that long-term COVID-19 symptoms and sequelae may disproportionately impact racialized groups for some of the same reasons that they experience greater rates of infection, illness severity, and death—lack of access to high-quality care and difficulty persuading providers that their experiences and conditions are real [23].

During the COVID-19 pandemic, efforts to slow the spread of COVID-19 and mitigate its adverse consequences have led to a remarkable transformation in U.S. healthcare delivery [10, 24]. As early as March 2020, the Centers for Disease Control and Prevention (CDC) called for a prioritization of telehealth care, and at both the state and federal levels reimbursement for telehealth services was markedly expanded [25, 26]. Prior to the COVID-19 pandemic, health systems reported relatively low rates of telehealth use [27], and even those with high adoption performed fewer than 100 virtual visits per day [28]. Now, many health systems conduct hundreds of virtual visits per day [29] and telehealth is considered a key access point for diagnosis, triage, and treatment of health conditions [10, 29, 30]. With this unprecedented increase in telehealth adoption, pre-pandemic care disparities that existed during a time when this care modality was largely unavailable may be reduced or mitigated.

Most early pandemic research has found disparities in access to telehealth for some groups, including groups disproportionately impacted by COVID-19 disease. One study by Chunara, Zhao, Lawrence, et al. [31] showed that from March 19 to April 20, 2020, Black patients in a large healthcare system in New York City had significantly lower odds of any telehealth use (virtual urgent and ambulatory encounters, together) compared with White patients. Pierce and Stevermer [32] found that during a similar time period among Medicare, Medicaid, self-pay, and privately insured patients, compared with White patients and urban patients, Black patients and rural patients in a Missouri academic medical center were less likely to use family medicine telehealth care. For those that did, Black patients were less likely than White patients to have access to audio-video (versus audio-only) family medicine telehealth. In addition, in the first year of the pandemic, individuals across the U.S. with employer-sponsored insurance living in high poverty and rural areas had the smallest increases in telehealth use of any type according to a study by Cantor, McBain, Pera et al. [33]. Conversely, research on a healthcare organization in Southern California serving racially/ethnically and socio-economically diverse patients found that compared with pre-pandemic levels, increases in synchronous telephone or live-video-audio telehealth use of any kind during the pandemic’s first year were largest among Hispanic and low-income patients [34].

### Motivation for this study

With adequate access to high-quality primary care, morbidity, adverse clinical outcomes, and negative social consequences (e.g., loss of income and economic instability) such as those of long-term COVID-19 disease can be reduced [35–39]. Primary care providers who know their patients and are aware of their life circumstances are in ideal positions to act as care hubs, coordinating and personalizing COVID-19 recovery, providing referrals to specialty care as appropriate, and addressing barriers to needed services and supports [35]. During the COVID-19 pandemic, health systems have relied more heavily on telehealth to ensure access to these crucial primary care services [40]. Yet, access to telehealth care has not been distributed equitably across racialized groups, income levels, or geographic areas. That said, existing research on telehealth disparities during the pandemic has covered relatively small geographic regions and has been either cross-sectional in nature or short in duration. Moreover, it has largely focused on general patient populations rather than those with COVID-19—patients who may experience outsized benefit from enhanced access to primary care given the very real potential for long-term COVID-19 conditions and sequelae. In addition, existing research has neglected to examine virtual primary care specifically, an important access, triage, and treatment point for COVID-19 patients. In this study, we seek to fill some of these gaps by exploring differences in longer-term access to virtual and in-person primary care by race/ethnicity and community social vulnerability among patients diagnosed with COVID-19 in a large, multi-state health system during the first year and a half of the pandemic, with the goal of understanding in/equity of health system response to COVID-19 disease among a racially/ethnically and socio-demographically diverse group of COVID-19 patients.

## Methods

### Study aims

Using a retrospective, observational cohort design, we examined the association between race/ethnicity, community social vulnerability, and use of virtual primary care among adult patients diagnosed with COVID-19 between March and July 2020 at a Providence health system site, comparing virtual primary care use in the year prior to and the year after COVID-19 diagnosis. To explore whether disparities in access to virtual primary care may have been offset by improved access to in-person primary care, we compared concurrent use of in-person primary care in the year before and after COVID-19 diagnosis by race/ethnicity and community social vulnerability among the same patients.

### Study data and population

Data for this study came from the Providence health system electronic health record (EHR), which contains information on patient demographic and health characteristics, and healthcare utilization. Providence is a non-profit healthcare system operating across seven states: Alaska, California, Montana, New Mexico, Oregon, Texas, and Washington [41]. Individuals were included in the study if they were at least 18 years of age at the time of their COVID-19 diagnosis, if they did not die in the year following their COVID-19 diagnosis, and if they resided in a state served by Providence. Individuals were excluded from the study if they resided in Texas (*n* = 46); at the time of this study, the Providence Texas EHR was not fully integrated with the larger Providence EHR and therefore complete data were not available for Texas’ patients. Individuals were also excluded from the study if they had missing community social vulnerability (*n* = 31) or race/ethnicity (*n* = 1072) information, resulting in a final study sample comprised of 11,326 individuals.

### Variables

#### Dependent variables

Our dependent variables included two binary variables for whether an individual used virtual primary care and in-person primary care, defined as having at least one virtual or in-person primary care visit, respectively. Primary care visits were identified in the EHR as visits that occurred with any provider delivering care in an outpatient department group designated as primary care. Visits were classified as virtual if they took place in a virtual office setting, or in-person if they took place in an in-person outpatient setting. A virtual office visit could include visits in which patients had audio-video or audio-only capability. Asynchronous messaging was not included in our definition of virtual primary care visits. In order to examine longer-range access to primary care (versus COVID-19 onset-related access), we censored visits that took place during the first 30 days after COVID-19 diagnosis when constructing each of our utilization outcomes, as these visits may have been associated with acute COVID-19 disease-related care.

#### Independent variables

Data on study participants’ race and ethnicity was used to create a mutually exclusive race/ethnicity variable comprised of seven categories: non-Hispanic White, non-Hispanic Black, non-Hispanic Asian, non-Hispanic American Indian/Alaskan Native (AI/AN), non-Hispanic Native Hawaiian/Pacific Islander (NH/PI), non-Hispanic other, and Hispanic/Latino. If a study participant was recorded as being Hispanic/Latino and any other race, they were categorized as Hispanic/Latino, otherwise they were categorized based on the most up-to-date race/ethnicity information available in the EHR.

Community social vulnerability was defined using the Social Vulnerability Index (SVI) from the CDC, which categorizes an area as socially vulnerable via factors such as poverty, lack of access to transportation, and crowded housing [42]. The SVI determines the social vulnerability of each census tract using a percentile ranking of the proportion of tracts that are equal to or lower in rank than the tract of interest along the SVI theme. For example, a tract ranking of 0.90 indicates that the tract of interest is more vulnerable than 90% of tracts in its state along the SVI theme in question [43]. The SVI ranks each tract on 15 social factors, which are then grouped into four themes: (1) socioeconomic status, (2) household composition and disability, (3) minority status and language, and (4) housing type and transportation [44]. Following the approach outlined by Flanagan, Gregory, Hallisey et al. [45], we created binary variables for each of the four SVI themes which were equal to 1 if an individual resided in a tract that was more vulnerable along the SVI theme in question than 90% or more of tracts in its state, and 0 if otherwise.

The post-COVID-19 diagnosis period was identified based on a study participant’s first positive COVID-19 diagnosis. From this we created a binary variable that identified whether an observation occurred before or after the COVID-19 diagnosis date.

Demographic variables included age group, calculated as the study participant’s age at the date of their COVID-19 diagnosis (categories included < 20, 20–34, 35–44, 45–54, 55–64, 65–74, and ≥ 75); and sex (categories included male and female). Health status variables included a proxy variable for COVID-19 severity, which was a variable indicating whether a study participant was diagnosed with COVID-19 in an inpatient healthcare setting (categories included yes, no); and five binary variables indicating whether a study participant had chronic conditions including diabetes, hypertension, coronary artery disease, chronic kidney disease, and/or congestive heart failure (categories included yes, no). These were the five most common chronic conditions among the study sample that were flagged by Providence as potentially leading to worse COVID-19 disease and outcomes, and are relevant to pre-COVID-19 disease primary care needs and utilization.

### Statistical analysis

#### Descriptive analysis

We examined demographic and health characteristics of our study sample overall and by virtual primary care use status by computing cell sizes and percentages for categorical variables. We descriptively examined primary care use outcomes by computing cell sizes and proportions of the study sample who used each type of primary care, and by computing means and standard deviations for each type of primary care during the study period overall, and separately for the year before and after COVID-19 diagnosis.

#### Regression analysis

We employed logistic regression and calculated average marginal effects to explore changes in the probabilities of each primary care outcome from the year before to the year after COVID-19 diagnosis by race/ethnicity and community social vulnerability. To do this, we created interaction terms for the post-COVID-19 period and each category of race/ethnicity, and interaction terms for the post-COVID-19 period and each binary SVI theme variable. Following the approach employed by other researchers studying COVID-19 and/or telehealth disparities [46–49], and because associations between race/ethnicity, community social vulnerability, and access to care reflect persistent structural racism and inequitable distribution of resources in the U.S., we examined both unadjusted differences in use of care, which we consider our main models, and adjusted differences that account for the demographic and health characteristics described above. To address potential heteroskedasticity, we estimated robust standard errors in all regression models [50]; statistical significance was determined at the traditional 5% alpha level. All analyses were performed using Stata version 14.2 [51]. This study was reviewed and approved by the Providence Institutional Review Board.

## Results

### Population characteristics

Table 1 provides demographic and health characteristics of the study sample overall and stratified by virtual primary care use status. Overall, the study population was largely comprised of Hispanic/Latino (41.7%) and non-Hispanic White (39.5%) individuals. Individuals who used virtual primary care during the study period were more likely to be non-Hispanic White, Black, and Asian. Over half the study sample (56.7%) lived in a census tract categorized as socially vulnerable along at least one community social vulnerability theme, with the housing type and transportation theme being the most common (41.6%). Individuals who used virtual primary care during the study period were less likely to live in tracts categorized as socially vulnerable along each of the community social vulnerability themes.

**Table 1:** Characteristics of study sample overall and by virtual primary care use status

Characteristic	Overall (*N* = 11,326) (n/%)	Virtual Primary Care Use	
		No (*n* = 9966)	Yes (*n* = 1360)
		(n/%)	(n/%)
**Race/Ethnicity**			
Non-Hispanic White	4471 (39.5%)	3874 (38.9%)	597 (43.9%)
Non-Hispanic Black	622 (5.5%)	509 (5.1%)	113 (8.3%)
Non-Hispanic Asian	605 (5.3%)	508 (5.1%)	97 (7.1%)
Non-Hispanic NH/PI	225 (2.0%)	198 (2.0%)	27 (2.0%)
Non-Hispanic AI/AN	92 (0.8%)	90 (0.9%)	2 (0.2%)
Non-Hispanic Other	592 (5.2%)	537 (5.4%)	55 (4.0%)
Hispanic/Latino	4719 (41.7%)	4250 (42.6%)	469 (34.5%)
**Community Social Vulnerability Theme**			
Any of the Four Themes	6420 (56.7%)	5761 (57.8%)	659 (48.5%)
Socioeconomic Status	2223 (19.6%)	2003 (20.1%)	220 (16.2%)
Household Composition	2502 (22.1%)	2242 (22.5%)	260 (19.1%)
Minority Status/Language	2656 (23.5%)	2400 (24.1%)	256 (18.8%)
Housing Type/Transportation	4716 (41.6%)	4236 (42.5%)	480 (35.3%)
**Age Group**			
< 20	267 (2.4%)	247 (2.5%)	20 (1.5%)
20–34	3191 (28.2%)	2880 (28.9%)	311 (22.9%)
35–44	1947 (17.2%)	1710 (17.2%)	237 (17.4%)
45–54	2021 (17.8%)	1758 (17.6%)	263 (19.3%)
55–64	1774 (15.7%)	1528 (15.3%)	246 (18.1%)
65–74	1098 (9.7%)	939 (9.4%)	159 (11.7%)
≥ 75	1028 (9.1%)	904 (9.1%)	124 (9.1%)
**Sex**			
Male	5411 (47.8%)	4932 (49.5%)	479 (35.2%)
Female	5915 (52.2%)	5034 (50.5%)	881 (64.8%)
**COVID-19 Diagnosis Setting**			
Inpatient	2715 (24.0%)	2535 (25.4%)	180 (13.2%)
Non-Inpatient	8611 (76.0%)	7431 (74.6%)	1180 (86.8%)
**Chronic Conditions**			
Hypertension	2536 (22.4%)	2284 (22.9%)	252 (18.5%)
Diabetes	1673 (14.8%)	1512 (15.2%)	161 (11.8%)
Coronary Artery Disease	1176 (10.4%)	1061 (10.7%)	115 (8.5%)
Chronic Kidney Disease	1046 (9.2%)	942 (9.5%)	104 (7.7%)
Congestive Heart Failure	546 (4.8%)	499 (5.0%)	47 (3.5%)

*Abbreviations*: *NH/PI* Native Hawaiian/Pacific Islander, *AI/AN* American Indian/Alaskan Native

With respect to the other demographic and health characteristics of the sample, the majority of individuals (81.3%) were under the age of 65, and there was an almost even split between male (47.8%) and female (52.2%) sex individuals. Older individuals and female sex individuals were more likely to use virtual primary care during the study period. About one quarter of individuals (24.0%) were diagnosed with COVID-19 in an inpatient setting, and about one in five individuals (22.4%) had hypertension; a smaller proportion had diabetes (14.8%), coronary artery disease (10.4%), chronic kidney disease (9.2%), and congestive heart failure (4.8%). Individuals who were diagnosed with COVID-19 in an outpatient setting were more likely to use virtual primary care during the study period, as were individuals without chronic health conditions.

### Primary care use

Table 2 provides descriptive data on virtual and in-person primary care use overall and stratified by the year before and after COVID-19 diagnosis. Overall, about one in ten individuals (12.0%) used virtual primary care and about one in three (27.4%) used in-person primary care during the study period. While the proportion of individuals who used in-person primary care remained similar before and after COVID-19 diagnosis (21.0 and 20.7%, respectively), the proportion who used virtual primary care increased substantially after COVID-19 diagnosis (3.6% before to 10.3% after COVID-19 diagnosis). The average number of virtual and in-person primary care visits remained similar from the year before to the year after COVID-19 diagnosis.

**Table 2:** Primary care use, overall and before and after COVID-19 diagnosis

	Any Visits (N/%)	Number of Visits (Mean/SD)
**Virtual Primary Care**		
Overall	1360 (12.0%)	0.3 (1.0)
Year Before COVID-19 Diagnosis	405 (3.6%)	0.1 (0.3)
Year After COVID-19 Diagnosis	1164 (10.3%)	0.2 (0.9)
**In-Person Primary Care**		
Overall	3106 (27.4%)	1.1 (2.5)
Year Before COVID-19 Diagnosis	2375 (21.0%)	0.6 (1.5)
Year After COVID-19 Diagnosis	2339 (20.7%)	0.5 (1.3)

In constructing each of our utilization outcomes we censored visits that took place during the first 30 days after COVID-19 diagnosis

### Differences in primary care use by race/ethnicity

Table 3 gives the average marginal effects (percentage-point differences) for the probability of using each type of primary care for our main and ancillary regression models. In our main model that included race/ethnicity, community social vulnerability, and interactions for the post-COVID-19 period, differences in the likelihood of using virtual primary care were observed by race/ethnicity. For example, in the year prior to COVID-19 diagnosis, non-Hispanic Black individuals had an increased likelihood of using virtual primary care compared with non-Hispanic White individuals [3.30 percentage points (pp), *p* < 0.05], while individuals identified as non-Hispanic other and Hispanic/Latino race/ethnicity had lower likelihoods of using virtual primary care in the year prior to COVID-19 diagnosis (─2.53 pp., *p* < 0.05, and ─2.11 pp., *p* < 0.01, respectively). None of the interaction terms for time period and race/ethnicity were statistically significant, indicating that these differences in virtual primary care use by race/ethnicity persisted in the year following COVID-19 diagnosis. (Differences in virtual primary care use could not be assessed for the non-Hispanic AI/AN group due to small cell size.)

**Table 3:** Association between race/ethnicity, community social vulnerability, and primary care use among individuals diagnosed with COVID-19

	Virtual Primary Care Use		In-Person Primary Care Use	
	Main Model	Ancillary Model	Main Model	Ancillary Model
Post-COVID-19 Period	0.0689***	0.0691***	− 0.0161	− 0.0162
**Race/Ethnicity (reference Non-Hispanic White)**				
Non-Hispanic Black	0.0330*	0.0376*	− 0.0065	0.0097
Non-Hispanic Asian	0.0031	0.0023	0.0158	0.0175
Non-Hispanic NH/PI	0.0063	0.0078	− 0.0683**	− 0.0530*
Non-Hispanic AI/AN	–	–	− 0.0949**	− 0.0933**
Non-Hispanic Other	−0.0253*	− 0.0233	− 0.0695***	− 0.0623***
Hispanic/Latino	− 0.0211**	− 0.0196*	− 0.0601***	− 0.0510***
**Interactions for Race/Ethnicity & the Post-COVID-19 Period**				
Non-Hispanic Black*Post-COVID-19	− 0.0062	− 0.0060	0.0206	0.0208
Non-Hispanic Asian* Post-COVID-19	0.0141	0.0143	0.0256	0.0258
Non-Hispanic NH/PI* Post-COVID-19	− 0.0247	− 0.0248	0.0357	0.0352
Non-Hispanic AI/AN* Post-COVID-19	–	–	− 0.0026	− 0.0028
Non-Hispanic Other* Post-COVID-19	0.0035	0.0032	0.0530	0.0528
Hispanic/Latino* Post-COVID-19	0.0070	0.0067	0.0133	0.0134
**Community Social Vulnerability Theme**				
Socioeconomic Status Theme	0.0003	− 0.0002	0.0021	0.0028
Household Composition Theme	− 0.0101	− 0.0092	− 0.0177	− 0.0174
Minority Status/Language Theme	− 0.0083	− 0.0086	− 0.0372**	− 0.0373**
Housing Type/Transportation Theme	− 0.0036	0.0007	− 0.0561***	− 0.0435***
**Interactions for Community Social Vulnerability & the Post-COVID-19 Period**				
Socioeconomic Status*Post-COVID-19	0.0153	0.0157	0.0126	0.0128
Household Composition*Post-COVID-19	− 0.0025	− 0.0026	0.0116	0.0118
Minority Status/Language *Post-COVID-19	− 0.0029	− 0.0034	0.0055	0.0051
Housing Type/Transportation *Post-COVID-19	− 0.0146	− 0.0147	− 0.0080	− 0.0079
**Age Group (reference** 75**+)**				
< 20	–	− 0.0490***	–	− 0.1644***
20–34	–	− 0.0412***	–	− 0.1719***
35–44	–	− 0.0235*	–	− 0.1186***
45–54	–	− 0.0137	–	− 0.0758***
55–64	–	− 0.0060	–	− 0.0619***
65–74	–	0.0115	–	0.0160
Male Sex	–	−0.0360***	–	−0.0563***
Inpatient COVID-19 Diagnosis	–	− 0.0517***	–	− 0.1564***
Diabetes	–	0.0001	–	− 0.0241**
Hypertension	–	− 0.0092	–	− 0.0377***
Coronary Artery Disease	–	0.0107	–	0.0122
Chronic Kidney Disease	–	0.0206*	–	0.0317*
Congestive Heart Failure	–	− 0.0056	–	0.0075
N	22,652	22,652	22,652	22,652

Average marginal effects for factor levels computed based on discrete change from reference level. Robust standard errors calculated using sandwich estimator. In constructing each of our utilization outcomes we censored visits that took place during the first 30 days after COVID-19 diagnosis

*Abbreviations*: *NH/PI* Native Hawaiian/Pacific Islander, *AI/AN* American Indian/Alaskan Native

^*^p < 0.05

^**^p < 0.01

^***^p < 0.001

Similar to the differences identified in virtual care utilization, in the year prior to COVID-19 diagnosis, there were significant differences in the likelihood of use of in-person primary care by race/ethnicity, with individuals identifying as non-Hispanic AI/AN (─6.83 pp., *p* < 0.01), non-Hispanic NH/PI (─9.49 pp., *p* < 0.01), non-Hispanic other (─6.95 pp., *p* < 0.001), and Hispanic/Latino (6.01 pp., *p* < 0.001) race/ethnicity having lower likelihoods of using in-person primary care compared with non-Hispanic White individuals. Again, none of the interaction terms were significant, indicating that these differences persisted in the year after COVID-19 diagnosis.

In ancillary models that adjusted for demographic and health characteristics, nearly all differences in virtual and in-person primary care use by race/ethnicity were similar in magnitude and significance. The only difference that was slightly reduced was the difference in virtual primary care use for non-Hispanic other race/ethnicity individuals (from ─2.53 pp., *p* < 0.05, to ─2.33 pp., *p* = 0.058).

### Differences in primary care use by community social vulnerability

In our main models that included community social vulnerability, race/ethnicity, and interactions for the post-COVID-19 period, use of virtual primary care was not significantly different by community social vulnerability in either the year before or after COVID-19 diagnosis. However, differences in use of in-person primary care by community social vulnerability were observed. For example, individuals residing in areas categorized as vulnerable based on minority status and language, and housing type and transportation had decreased likelihoods of using in-person primary care in the year before COVID-19 diagnosis (─3.72 pp., *p* < 0.01, and ─5.61 pp., *p* < 0.001, respectively). None of the interaction terms for time period and community social vulnerability theme were significant, indicating that these differences persisted in the year after COVID-19 diagnosis. In ancillary models, differences in in-person primary care use by community social vulnerability were similar in magnitude and significance.

## Discussion

Our study is one of the first to examine disparities in virtual and in-person primary care use among individuals diagnosed with COVID-19. Findings from our research suggest that, although there was a substantial increase in access to virtual primary care in the wake of the initial wave of COVID-19 infections and telehealth expansions, this did not substantially alter pre-pandemic disparities in access to primary care. Overall, only 12% and 27% of individuals diagnosed with COVID-19 used any virtual and in-person primary care, respectively, during the two-year study period. Disparities in use of virtual primary care were observed by race/ethnicity, and for some racialized groups, may have been compounded by concurrent disparities in use of in-person primary care. Specifically, Hispanic/Latino and non-Hispanic other race/ethnicity individuals were less likely to use virtual *and* in-person primary care compared with non-Hispanic White individuals in the year prior to COVID-19 diagnosis, and these disparities persisted in the year following COVID-19 diagnosis. Individuals who identified as Non-Hispanic NH/PI were no more likely to use virtual primary care compared with non-Hispanic White individuals, and were less likely to use in-person primary care. On the other hand, we found that during the study period, non-Hispanic Black individuals were more likely to use virtual primary care than non-Hispanic White individuals. These differences were observed when accounting for community social vulnerability and after controlling for demographic and health characteristics.

Previous research on racial and ethnic disparities in access to virtual care during the pandemic provides mixed results. For example, some studies have found that Black individuals had less access to urgent and ambulatory telehealth encounters and family medicine telehealth visits, compared with White individuals [31, 32], while others have found that Black individuals were more likely than White individuals to self-report using telehealth generally because of the pandemic [52]. However, previous studies examined shorter time periods or were narrower in geographic focus. Moreover, previous studies included general patient populations rather than those diagnosed with COVID-19, making direct comparisons to our own findings challenging. In addition, they did not distinguish between Hispanic and non-Hispanic Black patients, and most did not distinguish types of virtual care use (e.g., primary care versus telehealth care more broadly), further compounding comparison issues. That said, our study adds to the growing body of evidence demonstrating that White, non-Hispanic individuals generally enjoyed greater access to virtual care than non-White and Hispanic/Latino individuals during the COVID-19 pandemic—a particularly concerning finding given that racialized groups generally experience greater chronic disease burden than White and non-Hispanic populations [53] and increased COVID-19 infection rates, both of which may necessitate increased access to care. In addition, our study highlights what pre-pandemic research has shown: that racial/ethnic disparities in access to virtual care have existed since the advent of telehealth care, and rather than improving disparities through expansion of telehealth, the pandemic has only exacerbated and/or shed more light on them [48, 49, 54, 55].

Overall, we found no significant differences in use of virtual primary care by community social vulnerability. Yet, we did observe disparities in the use of in-person primary care that were not improved by increased access to virtual care. Specifically, regression analysis revealed that individuals living in areas characterized as vulnerable based on minority status and language, and housing type and transportation were less likely to use in-person primary care and no more likely to use virtual primary care than individuals living in areas not characterized as vulnerable in these ways. These findings held in both models that adjusted only for race/ethnicity and in models that additionally adjusted for age, sex, and chronic conditions.

To the best of our knowledge, previous studies have not used the CDC’s Social Vulnerability Index to explore disparities in access to virtual care during the pandemic. However, existing studies have examined factors related to community social vulnerability, including increased poverty and rural geography, finding pandemic-related disparities in access to virtual care along these dimensions [32–34]. In addition, evidence has clearly demonstrated how mechanisms of social stratification at the community level (e.g., segregation, community disinvestment, geographical concentration of poverty) result in differential access to care, and that community characteristics directly and indirectly shape what and where care is available, and the quality of that care [56]. Our findings support and add to existing literature on the association between community-level vulnerability and access to care, indicating that even after accounting for race/ethnicity and controlling for demographic and health characteristics, those living in areas characterized by greater rates of non-White and non-English speaking individuals, and by crowded housing and lack of transportation, experienced disparate access to primary care prior to and during the first year and a half of the pandemic.

Taken together, our findings highlight the urgent need to ensure equitable access to virtual (and in-person) primary care. This is particularly important given additional surges in cases and the emergence of new COVID-19 variants, all of which signal that the pandemic is not likely to end soon and that virtual care will remain an important care modality. Furthermore, there is a growing “care debt” that has the potential to lead to deleterious downstream consequences such as complications from unmanaged health conditions and incapacitation of an already overwhelmed healthcare system [29]. To create health systems that can effectively manage these contingencies, it will be crucial to transition telehealth services from a crisis intervention tool to an equitable and sustainable system for providing proactive patient care.

Evidence-based strategies exist for creating more equitable telehealth and primary care infrastructure [31]. First, conducting targeted patient outreach and actively connecting with individuals and groups who experience barriers to care has been shown to improve access to and utilization of care [57]. For example, in a study by Ospina-Pinillos et al. [58], participatory design methods were used to tailor the website of a virtual mental health clinic to improve outreach to Spanish-speaking individuals, which led to adequate acceptability levels in the website’s homepage, and triage, booking, and video visit systems for Spanish-speakers, and also enabled the clinic to identify the need for tailored assessment tools and greater integration with Spanish-speaking services and communities. In addition, a systematic review of interventions aimed at modifying the healthcare system to better outreach to and serve racialized groups and communities revealed that these interventions were associated with both improved processes of care delivery and reduced access disparities [59]. However, health systems must first be able to identify those experiencing barriers to care and find ways to create meaningful connections with them. This necessitates leveraging our current understanding of the multiple intersecting individual and community factors affecting access to care and addressing them in outreach materials and methods. Furthermore, at a systems level, this means deconstructing current systems which are inherently racist, overtly discriminatory, and implicitly biased, and rebuilding them into more just and healing systems that are acceptable and comfortable for diverse patient populations. For virtual care, this also means conducting additional research on what constitutes effective and trustworthy outreach and communication to diverse populations [31].

Next, developing representative provider networks can improve capacity of and access to care, while at the same time improving quality of care for underserved individuals and communities. Racial/ethnic concordance between patients and providers is associated with improved use of preventive services, satisfaction with care, patient-provider communication quality, and patient participation in care and decision-making [60, 61]. In addition, evidence shows that clinicians from racialized groups are more likely to treat patients from racialized groups, including those who live in medically underserved and vulnerable areas [62]. However, policymakers and health systems must purposefully devote financial and other resources to improving provider representativeness and dismantling racist and discriminatory practices including those that have resulted in a current provider supply that is more White and socioeconomically advantaged than the general U.S. population [63].

Designing culturally appropriate tools and technology that enable and improve access requires adaptations to systems predominantly designed for White, English-speaking individuals. To that end, health systems can collect and incorporate input on telehealth tools and technologies from racialized groups and those with limited English proficiency [64], as evidence indicates that cultural and linguistic tailoring can improve healthcare access and outcomes [58, 65–67]. Data collection and user testing should be done in a participatory manner in which cultural adaptations, and knowledge and language translation are co-designed with patients and/or research participants [58]. Health systems can also increase robust adoption of the National Culturally and Linguistically Appropriate Services Standards developed by the U.S. Department of Health and Human Services [68], which are intended to provide health workers and systems with a blueprint for developing equitable, understandable, respectful systems of care.

Policy solutions are also needed to address systemic barriers to care, such as inequitable distribution of healthcare and enabling resources. A recent survey found that limited broadband connectivity and related technology (e.g., computers and smart phones) has created barriers to telehealth during the pandemic [69]. This issue has particularly impacted individuals in rural areas and those over the age of 65. One policy solution is to provide funding for broadband expansion in medically underserved communities. Several initiatives are underway to accomplish this: As part of the American Rescue Plan Act of 2021, the Federal Communications Commission is launching the $3.2 billion Emergency Broadband Benefit program to help Americans with qualifying household incomes obtain high-speed internet [70]. In addition, a $100 million federal pilot program has been implemented to cover eligible costs of broadband connectivity, network equipment, and information services needed to provide connected care services to patients; and the COVID-19 Telehealth Program included $200 million in Congressional appropriations to help healthcare providers provide connected care to patients at their homes or in mobile locations [71, 72]. Time and future research will tell whether these policy solutions have reduced disparities in access to telehealth care.

Other policy and systems-level solutions that have been shown to improve access to primary care among underserved populations and communities include expanding scope of practice laws for and increasing the use of non-physician clinicians; expanding the supply of non-hospital-based clinics such as Federally Qualified Health Centers (FQHCs); increasing the availability of after-hours primary care services; and removing cost-related barriers to primary care such as cost-sharing [73].

### Limitations

This study has some limitations worth noting. First, our study sample is limited to Providence patients in six mostly Mid−/Western U.S. states, which may limit generalizability to Southern and North−/Eastern states. That said, this study provides data on patients across a large, multi-state geographic area that includes both rural and urban areas, enhancing generalizability compared with existing research on smaller geographic areas and largely urban centers. Next, our sample is comprised of patients who tested positive for COVID-19, yet evidence has demonstrated disparities in COVID-19 testing rates among racialized groups and those with limited English proficiency, even as they experience higher COVID-19 infection rates [74–76]. Therefore, our sample likely does not include all Providence patients who contracted COVID-19. If patients from racialized groups who contracted COVID-19 were tested at a lesser rate than non-Hispanic white patients, our results likely underestimate disparities in access to care. Despite this, the fact that our COVID-19 positive sample was largely comprised of Hispanic/Latino patients while the larger Providence patient population is primarily comprised of non-Hispanic white patients enhances confidence in our findings. Finally, various issues arise in analyses of electronic health record data and should be taken into consideration when interpreting our findings. For example, if Providence patients received care outside of a Providence setting, it is not recorded in the EHR or included in our analyses. In addition, the EHR data does not contain information on other relevant factors such as socio-economic status or access to enabling resources. However, we did include census tract-level socioeconomic and resource-related variables via the SVI, and thus captured at least some of the variability in these factors and their association with access to care.

## Conclusion

The pandemic has further illuminated the persistent inequities that lead to poorer access to care and health outcomes among racialized groups and vulnerable communities. Our study adds to the mounting body of evidence that lays bare these inequities. Using data from a large health system across multiple states, we found disparities in utilization of virtual and in-person primary care by both race/ethnicity and community social vulnerability among individuals diagnosed with COVID-19, some of the same groups of people who have been hit hardest by COVID-19 infections, morbidity, economic consequences, and mortality. The importance of primary care, together with widespread telehealth expansion brought about by the COVID-19 pandemic highlight both an urgent need and unprecedented opportunity to address these disparities, but only if solutions are purposefully designed and implemented to address their root causes [31, 34].

## Data Availability

The dataset used in this study was derived from electronic medical records and includes individual-level identifiers and protected health information that are not publicly available due to human subjects protection requirements. We are unable to provide this dataset for public use. However, requests for summary information may be submitted to the corresponding author for consideration.

## Abbreviations

AI/AN: American Indian / Alaska Native
CDC: Centers for Disease Control and Prevention
COVID-19: Coronavirus Disease 2019
EHR: Electronic Health Record
NH/PI: Native Hawaiian / Pacific Islander
SVI: Social Vulnerability Index
U.S.: United States
